# Atmospheric chemistry of bioaerosols: heterogeneous and multiphase reactions with atmospheric oxidants and other trace gases

**DOI:** 10.1039/c6sc02353c

**Published:** 2016-07-28

**Authors:** Armando D. Estillore, Jonathan V. Trueblood, Vicki H. Grassian

**Affiliations:** a Department of Chemistry & Biochemistry , University of California San Diego , La Jolla , California 92093 , USA . Email: vhgrassian@ucsd.edu ; Fax: +1-858-534-6255 ; Tel: +1-858-534-2499; b Scripps Institution of Oceanography and Department of Nanoengineering , University of California San Diego , La Jolla , California 92093 , USA

## Abstract

Advances in analytical techniques and instrumentation have now established methods for detecting, quantifying, and identifying the chemical and microbial constituents of particulate matter in the atmosphere. For example, recent cryo-TEM studies of sea spray have identified whole bacteria and viruses ejected from ocean seawater into air. A focal point of this perspective is directed towards the reactivity of aerosol particles of biological origin with oxidants (OH, NO_3_, and O_3_) present in the atmosphere. Complementary information on the reactivity of aerosol particles is obtained from field investigations and laboratory studies. Laboratory studies of different types of biologically-derived particles offer important information related to their impacts on the local and global environment. These studies can also unravel a range of different chemistries and reactivity afforded by the complexity and diversity of the chemical make-up of these particles. Laboratory experiments as the ones reviewed herein can elucidate the chemistry of biological aerosols.

## Abstract 2

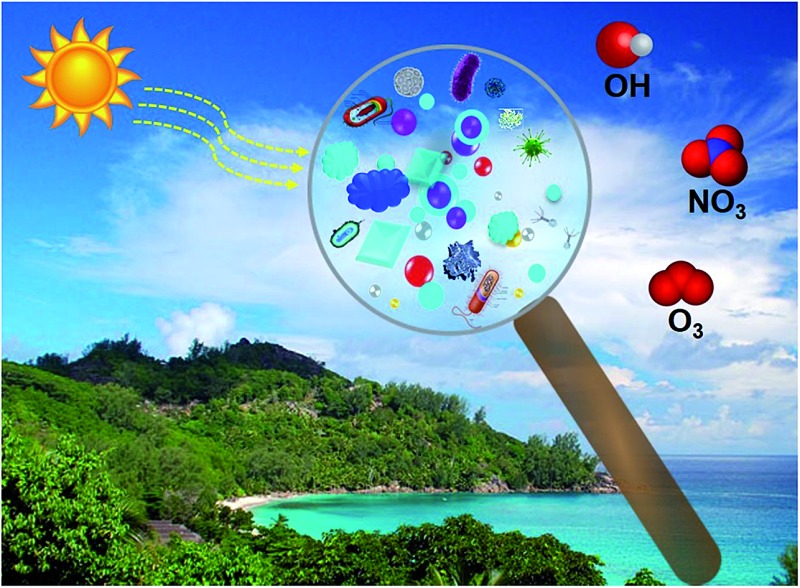
Once airborne, biologically-derived aerosol particles are prone to reaction with various atmospheric oxidants such as OH, NO_3_, and O_3_.

## Introduction

Generated from various sources, aerosols are ubiquitous in the atmosphere and play a crucial role in global climate, urban visibility, and human health. These airborne particles, ranging in size from a few nanometers (nm) to tens of micrometers (μm), can be released from primary sources such as industrial processes, biomass burning, volcanic emissions, or sea spray.^1–5^ In addition, aerosol particles can nucleate from atmospheric gases to form secondary organic aerosol (SOA).^6^ SOA is composed of a myriad of complex chemical compounds exhibiting a wide range of molecular structures and phase states which influences their physicochemical properties and reactivities.^7,8^ Atmospheric aerosols are strongly related to a plethora of health concerns as they are respiratory irritants, can cause cardiovascular problems, and are linked to increased mortality.^9–13^ Furthermore, depending on their chemical composition, aerosols can absorb or scatter solar and terrestrial radiation and can act as cloud condensation and ice nuclei which in turn plays a major role in global temperatures and precipitation.^14^

A major source of primary aerosol organic matter, ranging from nm to μm in size, in the atmosphere is primary biological organic aerosol particles (PBAP) that are emitted directly from the biosphere to the atmosphere.^15^ These particles are comprised of dead and live cells, cell fragments, pollen (>10 μm), bacteria (∼1 μm), fungi, algae, moss and fern spores (∼10 μm), viruses (<0.1 μm), and other fragments of animals and plants.^15–19^ Studies on exposure to these particles are associated with a variety of health issues with major public health impacts, including infectious diseases, acute toxic effects, allergies and cancer.^20,21^ Collective efforts are actively pursued to decode the intricate details of the interaction of the coexisting population of microbiomes and nanobiomes of human, plants, the earth, the ocean and the atmosphere from molecular to global scale.^22,23^ As an example, the diversity of the atmospheric microbiome is severely affected by dust events^24^ and tropical storms.^25^ The relative abundance of desert soil-associated bacteria has been shown to increase during dust events, while the relative abundance of anthropogenic-influenced taxa decreases.^24^

In addition, biologically-derived compounds are known to be present and are incorporated in other primary aerosol particles such as sea spray aerosol (SSA) and dust particles that contribute to aerosol mass.^26–30^ For example, amino acids in both free and combined forms (*i.e.*, peptides and proteins) are an important component in SSA^31–33^ as well as a range of terrestrial bioaerosol.^34^ They are the most abundant group of pollen allergens^35^ and are found in both coarse biological particles such as pollen grains (>10 μm) as well as the fine fraction of air particulate matter (<2.5 μm). In addition to being an important component of particulate aerosol, free and combined forms of amino acids have been found in hydrometeors^36^ and rain.^37^

**Fig. 1:**
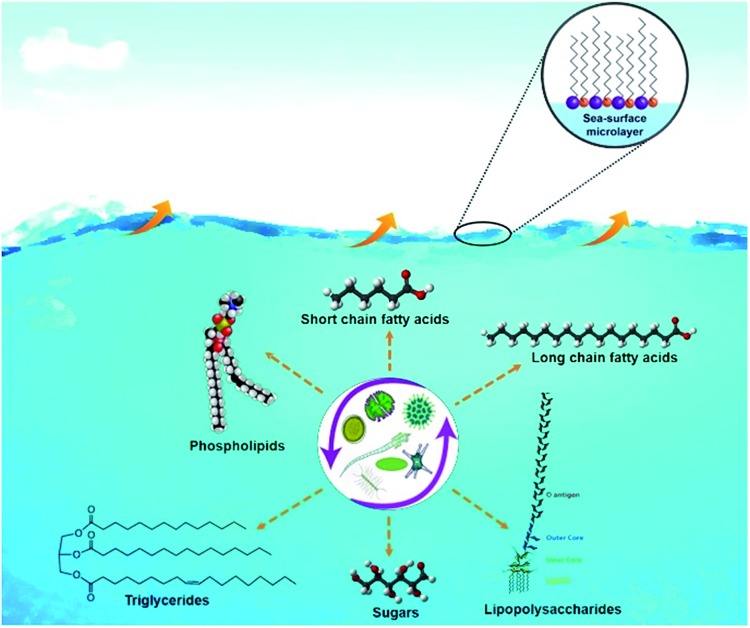
Oceans, covering >70% of Earth's surface, are a major source of biologically-derived compounds in the atmosphere. The biological, chemical, and physical processes in oceans affects the major chemical composition of sea-spray aerosol emitted into the atmosphere.

**Fig. 2:**
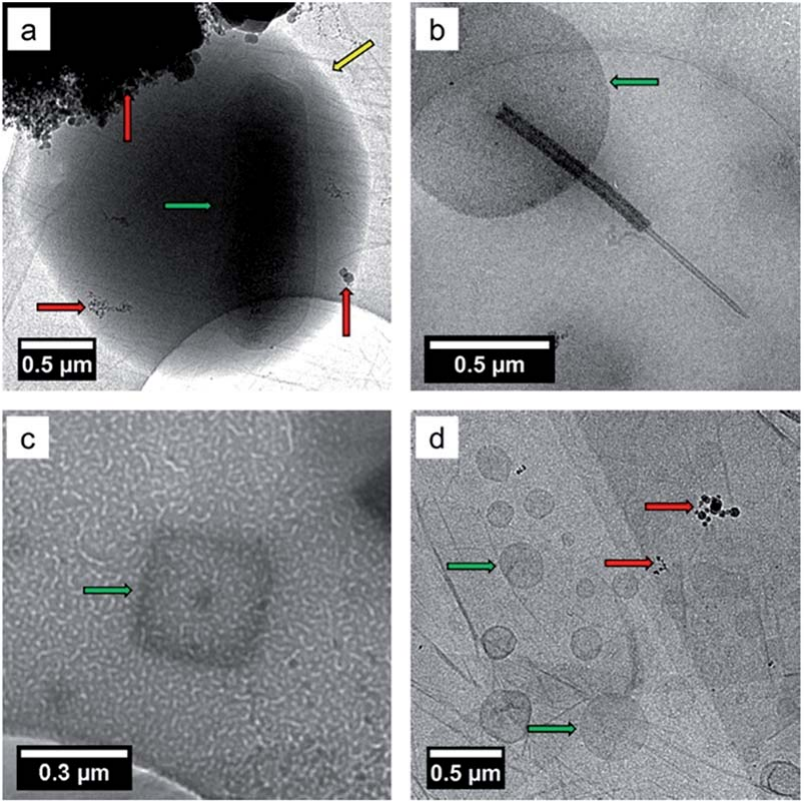
Bright field TEM images of SSA prepared by cryo-TEM showing (a) a whole bacterium inside a wet SSA droplet, (b) a diatom, (c) a virus particle, and (d) marine membrane vesicles. Yellow arrows indicate the edge of the SSA, the red arrows indicate contamination from the cryo-TEM preparation, and the green arrows indicate the biological particles. Adapted with permission from Patterson *et al.*, *ACS Cent. Sci.*, 2016, **2**, 40–47.

Recent studies showed that oceans, which cover >70% of earth's surface, are a major source of bacteria, viruses and biologically-derived compounds to the atmosphere. The interaction of ocean-atmosphere is schematically illustrated in Fig. 1. In a study conducted using a unique ocean-atmosphere facility containing 3400 gallons of natural seawater, Wang *et al.* ^38^ generated two successive phytoplankton blooms producing SSA with different composition and properties. In the first bloom, aliphatic-rich organics were present in submicron SSA that followed the abundance of phytoplankton measured by chlorophyll-a concentrations. During the second bloom, no organic enrichment was found in the particles collected. The studies explained the key link between biological, chemical, and physical processes occurring in the ocean and the resulting physicochemical properties of SSA particles.^38^ Furthermore, using cryogenic transmission electron microscopy (cryo-TEM) Patterson *et al.*,^27^ recently reported the detection of whole hydrated bacteria, diatoms, virus particles, marine vesicles from SSA samples as shown in Fig. 2.

Overall, it is believed that primary biological aerosol particles make up a sizable fraction of the aerosol budget, with 1000 Tg per year emitted in comparison to 3300 Tg per year for sea salt and 2000 Tg per year for mineral dust.^34^ Interest in biological aerosol particle research is rapidly growing due in part to the continued progress and availability of sophisticated analytical instrumentation. Primary biological organic aerosol has been identified, characterized and quantified in several field studies.^15,39,40^ Bioaerosols are detected using several analytical techniques which includes electron impact ionization aerosol mass spectrometry,^41,42^ fluorescence spectroscopy,^43,44^ and Raman spectroscopy,^45,46^ among others. For additional information on bioaerosol detection, we refer the readers to several excellent reviews recently published.^15,47–49^

Aerosols evolve away from their native states as they undergo heterogeneous and multiphase reactions that modify gas and particle phases,^50–55^ affecting both atmospheric composition and air quality.^50^ Tremendous progress has been made in the fundamental understanding of heterogeneous chemical reactions involving primary aerosols such as mineral dust^56,57^ and sea-spray aerosol.^33,58^ Similarly, great strides have been made through the many studies on secondary organic aerosol formation.^8,54,59–61^ Information gathered from these studies has made huge impacts in understanding the chemistry and properties of atmospheric aerosols. However equivalent investigations on biological aerosol and how they are chemically transformed in the atmosphere remains limited. The importance of studying the reactivity of PBAP are several fold, both practical as well as elementary in nature. These studies cover many areas of chemistry, biology, clinical medicine, and even biodefense. These biologically derived particles are fundamentally different from inorganic aerosols; potentially more interesting and certainly much more complicated as these particles come in varying size and shape, carry different molecular functional groups and consist of different conformations. Because of their complexity, PBAP respond differently to abrupt changes of environmental conditions in the atmosphere such as relative humidity (RH), temperature, and reactive trace gases. Furthermore, studies have shown that these particles play an important role as ice and cloud condensation nuclei.^38,62–68^

The focus of this article is to review some processes related to the reactivity of bioaerosols with emphasis on select oxidants; OH, O_3_, and NO_3_. We do not attempt to comprehensively review the entire field of bioaerosol reactivity, as this is a continuously evolving area of research. For simplicity, we use the term “bioaerosols” to refer to primary biological and biologically-derived aerosol particles. This review covers several sections on the reactivity with oxidants in the atmosphere including OH, O_3_ and NO_3_ as well as a section on the reactivity of other trace gases, new reaction pathways and photochemistry.

## Bioaerosol reactivity – oxidant chemistry and other reaction pathways

### A. OH reaction with bioaerosols and biologically derived compounds

The hydroxyl radical is one of the most reactive atmospheric oxidants and is known to be one of the reactive oxygen species (ROS) in atmospheric processes. Its interactions with gas-phase organic species and the underlying processes by such interactions have been investigated extensively.^59,69–71^ Biologically-derived molecules lofted into the atmosphere can be chemically and physically altered through reactive encounters with OH radicals in the daytime when their concentration is highest. For example, OH radicals can reactively modify saturated and unsaturated molecular moieties by causing H atom abstraction of the former and both H-atom abstraction from the alkyl chain and addition to the –C=C– of the latter.^72,73^ Damage to biological systems can be induced by direct attack of ROS such as OH radicals.^74^ Investigations on the interactions of proteins and amino acids with OH radicals reveal H atom abstraction from C–H bonds, either from the protein backbone or from an amino acid side chain. This H atom abstraction step represents the first step in the protein oxidation pathway.^75–77^ In an *in situ* mass spectrometric study of the interaction of OH radicals with glutathione (GSH), an antioxidant which is a cysteine-containing tripeptide in epithelial lining fluids (ELF), Enami *et al.*, showed that GSH are oxidized into sulfenic acid GSOH^–^, sulfinic acid GSO_2_H^–^, and sulfonic acid GSO_3_H^–^. This reaction oxidizes the GSH when inhaled particulates generate OH radical in the ELF *via* the Fenton-type chemistry.^78^

**Fig. 3:**
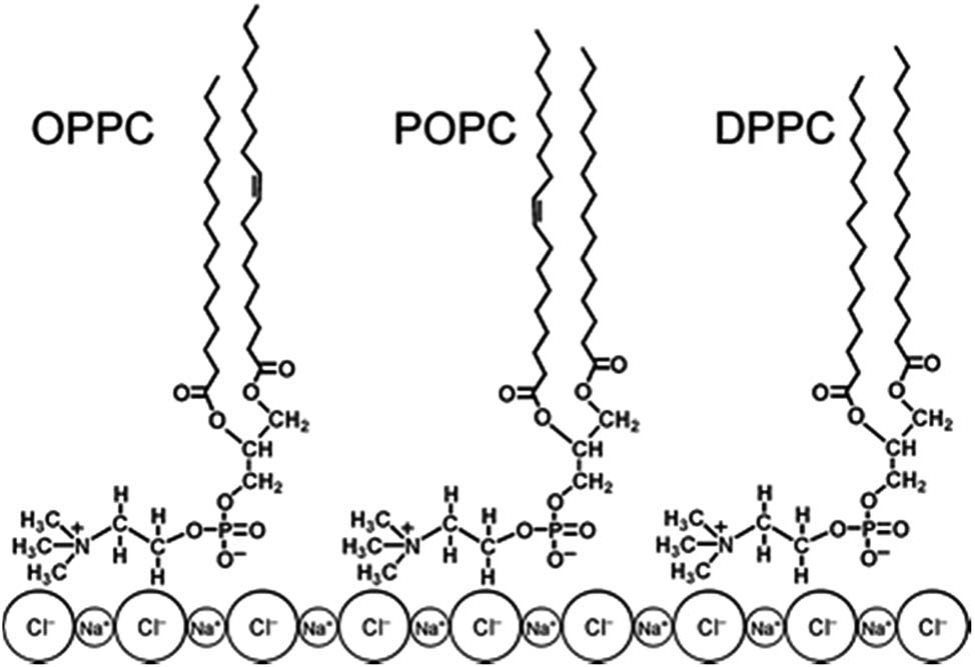
Structures of OPPC, POPC and DPPC, shown on the surface of solid NaCl. Adapted with permission from Dilbeck *et al.*, *Phys. Chem. Chem. Phys.*, 2013, **15**, 9833–9844.

Another class of molecules prone to reactive encounters in the atmosphere with OH radicals is phospholipids. Phospholipids are found at the sea surface microlayer (SSML), in the top most region of the ocean surface and in the atmosphere as an organic coating in sea-spray aerosol.^29,79–81^ Moreover, phospholipids in both the saturated and unsaturated forms are a major component in cell membranes.^82^ Finlayson-Pitts and co-workers performed experiments on the reaction of OH radical with 1,2-dipalmitoyl-*sn-glycero*-3-phosphocholine (DPPC) as a proxy for saturated and 1-palmitoyl-2-oleoyl-*sn-glycero*-3-phosphocholine (POPC) as proxy for unsaturated phospholipids on a surface of sodium chloride using diffuse reflection infrared Fourier transform spectrometry (DRIFTS) and matrix-assisted laser desorption/ionization-time-of-flight mass spectrometry (MALDI-TOF-MS) methods.^83,84^ Fig. 3 shows the structure of different phospholipids on the surface of NaCl as depicted in ref. 82, showing an “inverted micelle” where the hydrocarbon chains are exposed to the oxidizing environment.^85^ DPPC is a saturated phospholipid and thus only abstraction from the alkyl chain is possible, while both H atom abstraction and OH addition to the carbon–carbon double bond from the alkyl chains will occur in OPPC and its structural isomer POPC. In this “top down” experiment, the OH radical was generated in the gas phase over the phospholipid *via* photolysis of isopropyl nitrite in a mixture of N_2_ and air as shown in reactions (1)–(3):

R1 (CH_3_)_2_CHONO + *hν* → (CH_3_)_2_CHO˙ + NO

R2 (CH_3_)_2_CHO˙ + O_2_ → CH_3_COCH_3_ + HO_2_

R3 HO_2_ + NO → OH + NO_2_

The results of this experiment identified aldehydes, ketones, organic nitrates and nitrite ions as products of the reaction. The rate constant (*k*_1_) for the OH addition to the double bond in POPC was measured to be *k*_1_ > (3 ± 1) × 10^–13^ cm^3^ per molecule per s corresponding to a reaction probability, *γ*, for the addition reaction greater than (4 ± 1) × 10^–3^ while the rate constant (*k*_2_) for the DPPC–NaCl + OH is *k*_2_ = (7 ± 1) × 10^–14^ cm^3^ per molecule per s corresponding to *γ* of (8 ± 1) × 10^–4^ for the abstraction reaction.

Interestingly, unsaturated phospholipids such as OPPC can also be oxidized from the “bottom up” by the OH generated during the photolysis of nitrite ion in a NaNO_2_/NaCl salt mixture upon which OPPC is adsorbed, as shown in (R4). The presence of nitrite in the substrate is made possible by the displacement of chloride ion in SSA particles with nitrate, which in turn can photolyze readily to yield nitrite which then undergoes further reactions that yield OH radical as shown below.

R4 NO_2_^–^ + *hν* (290–400 nm) → NO + O^–^ + H_2_O → OH + OH^–^

Irradiation of the OPPC/NaNO_2_/NaCl mixture leads to the formation of organic nitrates and carbonyl compounds similar to the “top down” approach. In the absence of water vapor, carboxylate ions are also formed.^84,86^ These two different oxidation mechanisms of unsaturated phospholipids have important implications on the lifetime of the lipids. Freshly emitted SSA particles with phospholipid coating will experience top down oxidation. The onset of bottom up oxidation will soon set in when the SSA particles are exposed to species such as NO_2_^–^ and NO_3_^–^ that can be photolyzed to generate the OH oxidants. The organic nitrates generated as oxidation products of the two mechanisms can serve as reservoirs for oxides of nitrogen that can decompose to release NO_*x*_ back to the atmosphere.^87^

### B. NO_3_ reaction with bioaerosols and biologically derived compounds

Proteins are present in most biological systems at particularly high concentrations. Thus they are one of the most targeted components for oxidation in bioaerosols. To better access the activities of these aerosols, the valence electronic property of bioaerosols play a determinant role. The electronic properties of amino acid aerosols have been characterized which underlines the recent development on the instrumentation for characterizing bioaerosols as well as the advanced understanding on the properties of such types of bioaerosols. For example, Wilson *et al.* applied the VUV photoelectron imaging to study the electronic properties of glycine (Gly) and phenylalanine-glycine-glycine (Phe-Gly-Gly) in the dehydrated aerosol particle phase and determined the ionization energies and the molecular polarizabilities.^88^

Protein molecules that have been nitrated by polluted urban air can promote allergies upon their inhalation and deposition in the human respiratory tract.^89,90^ Several research investigations have shown that phenols and other aromatic compounds can be nitrated by gaseous air pollutants such as NO_2_ and NO_3_.^91–94^ Additionally, previous studies have shown that within biological systems, proteins can undergo a nitration reaction leading to the formation of 3-nitrotyrosine residues.^95,96^ In a key study done by Goschnick and Schuricht, it was found that the nitrogen content of pollen surfaces increased upon exposure to NO_2_.^97^ Working from this evidence, Franze *et al.* ^89,98^ first showed that the proteinaceous component of bioaerosols could also be nitrated when exposed to polluted urban outdoor air (*e.g.* vehicle exhaust) as well as synthetic gas mixtures containing H_2_O, NO_2_, and O_3_ at atmospherically relevant concentrations. The nitration reaction occurs by the addition of nitro groups to the aromatic ring of tyrosine residues in the polypeptide chain, resulting in the formation of 3-nitrotyrosine. Importantly, the extent of nitration was reduced under dry conditions, indicating that the hydration state of the aerosols could influence reaction pathways and kinetics. This process then can boost the power of existing allergens or make benign proteins allergenic. In their study, Franze and coworkers^89^ also collected samples of urban dust and found that up to 0.1% of the proteins in dust had been nitrated by traffic smog. Samples of allergenic proteins from birch pollen exposed to road junction in Munich, Germany found 10% of the proteins were nitrated and that number rose to 20% when exposed to smog in the laboratory. Interestingly, this study of pollutant–allergen interactions provided a molecular rationale for the medical reports which found an increase in allergic diseases in area with high traffic-related air pollution concentrations in combination with high NO_2_ and O_3_.^89,98^ The results were further corroborated by immunological experiments by Gruijthuijsen *et al.* who reported that allergenic potential of proteins are enhanced upon nitration.^99^ In a similar study, Rudich and co-workers^100^ conducted outdoor and laboratory exposure studies on the allergenicity of the allergenic mold *Aspergillus fumigatus*. The outdoor exposure experiments were performed in Modi'in, Israel. Their study demonstrated a dual effect of NO_2_/O_3_ pollution on the allergenic activity of *A. fumigatus* spore. Results showed that initial exposure of fungal spores leads to a 2–5 fold increase in allergenicity due to nitration compared to the control for short exposure times of up to 12 hours. At higher exposure times, the increase of allergenicity was less pronounced, which the authors ascribed to protein deamidation.^100^

**Fig. 4:**
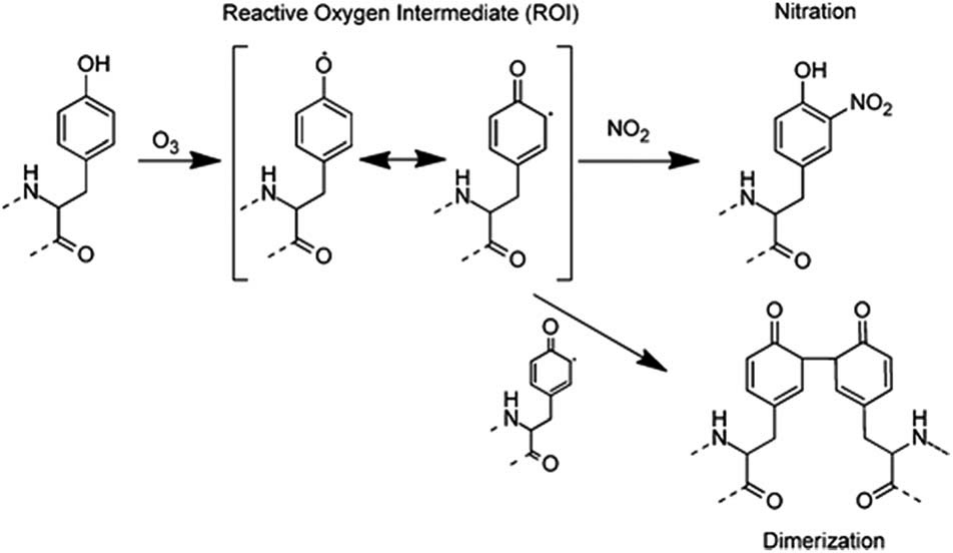
Schematic of a reaction pathway of residues within the protein BSA following exposure to ozone and followed by nitrogen dioxide. Adapted with permission from Shiraiwa *et al.*, *Environ. Sci. Technol.*, 2012, **46**, 6672–6680.

From these initial studies on protein nitration of bioaerosols in the atmosphere, several investigations then focused on the underlying kinetics and reaction mechanisms. Aiding such studies was the development of simple, fast, efficient, and inexpensive methods for the quantification of nitrotyrosine residues in protein molecules.^101–103^ Shiraiwa *et al.* ^104^ used a new kinetic modeling approach to show that the nitration of proteins with O_3_ and NO_3_ occurs through a two-step process involving the formation of long-lived reactive oxygen intermediates (ROIs) followed by nitration of the ROI *via* exposure to NO_2_. Shiraiwa *et al.* ^105^ then further studied the kinetics and reaction mechanism of protein nitration using an aerosol flow tube and the short lived radioactive tracer ^13^N in addition to a kinetic multilayer model. Their results further proved that the reactions proceed through the two-step mechanism as previously proposed by Shiraiwa^104^ *et al.* and as summarized in Fig. 4. In addition, it was found that the reaction of proteins on the surface of aerosol particles were rapidly nitrated, with the rate of nitration of the proteins in the interior of the particle being controlled by diffusion. In the absence of NO_2_, the ROIs were found to react with each other forming protein dimers. The efficiency and specificity of the protein nitration strongly depends on the nitrating agent and the reaction conditions. In another nitration study of the major birch pollen allergen Bet v 1, tetranitromethane was found to be the most efficient nitrating reagent, yielding nitration degree (ND) values of up to 70% over peroxynitrite (ONOO^–^) and O_3_/NO_2_ which yielded ND values up to ∼50 and ∼20%, respectively, with substantial amounts of side products from protein oxidation and degradation. Nitration rates were found to be higher for aqueous protein solutions (∼20% per day) than for solid or semisolid protein samples (∼2% per day).^106^

While it has been shown by Shiraiwa *et al.* that the principal reaction is a two-step process, the actual reaction mechanism and pathways still remained unclear as there are many reactive sites in tyrosine other than the phenolic site in which the first oxidation step could take place. Using quantum chemical methods, Sandhiya^107^ showed that the initial oxidation of tyrosine by O_3_ proceeds by H atom abstraction and addition reactions, leading to the formation of six different intermediates. The subsequent nitration reaction was then studied for all six intermediates in gas, aqueous, and lipid media. Their results showed that the nitration could affect both the side chain and aromatic ring of tyrosine, with some other resulting products formed from other reaction pathways.

Studies conducted by Wille *et al.* ^108–110^ on the reaction of nitrate radicals with aromatic amino acids demonstrated that the reaction leads to irreversible oxidative functionalization at the β-position or at the aromatic ring of the amino acids.^108,109^ In the case of aromatic dipeptides, in addition to oxidation at the ring, the oxidative damage of dipeptide linkage occurs in the aromatic N- and C-protected dipeptides.^110^ The results strongly suggest that this important atmospheric oxidant could potentially cause damage to peptides lining the respiratory tract and may contribute to pollution-derived diseases.

### C. O_3_ reaction with bioaerosols and biologically-derived compounds

The ozonolysis of unsaturated volatile organic compounds in the troposphere has been known to proceed *via* the Criegee intermediate (CI)^111^ and has been well studied in laboratory and theoretical investigations. The details of this reaction have been recently compiled in an excellent review article.^112^ Additionally, the interaction of ozone with organic aerosols, especially those containing unsaturated bonds, has been extensively studied.^55,113–117^

Karagulian and coworkers performed studies on the oxidation of OPPC adsorbed on NaCl as a model for lipids on sea salt using DRIFTS. Product identification was confirmed by matrix-assisted laser desorption/ionization (MALDI) mass spectrometry and Auger electron spectroscopy.^118^ Following exposure to O_3_, loss of the C=C functionality was observed with the subsequent formation of a stable secondary ozonide (1,2,4-trioxolane, SOZ) known to be formed in alkene–ozone reaction and confirmed by the strong band at ∼1110 cm^–1^. MALDI-TOF also confirmed the formation of SOZ at *m*/*z* = 809 and 831 attributed to SOZ adducts with H^+^ and Na^+^. The effect of water vapor on the ozonolysis reaction was also conducted at relative humidity (RH) ranging from 2 to 25%. These are relatively low RH values in order to maintain the phase state of NaCl as a solid to preserve the sample integrity for DRIFTS analysis. With increasing RH, the band at 1110 cm^–1^ that is associated with SOZ was dramatically decreased. This decrease of SOZ with hydration was more pronounced at the lower O_3_ concentration, which suggests competition of SOZ formation with the reaction of Criegee intermediate (CI) with water vapor. Vesna *et al.* suggested that, for the ozonolysis of arachidonic acid under humid conditions, the reaction of water with CI might open a pathway for the formation of smaller acids that lead to more significant changes in hygroscopicity.^119^ The authors also showed the increase of product yields of the ozonolysis of oleic acid with increasing RH.^120^

Dilbeck *et al.*,^121^ reported the ozone oxidation of 1-palmitoyl-2-oleoyl-*sn-glycero*-3-phosphocholine (POPC) adsorbed on salt mixtures using DRIFTS. Ozone reacts with the –C=C– backbone in the POPC *via* the Criegee mechanism. The experiments conducted at room temperature yielded secondary ozonide and a phospholipid aldehyde and carboxylic acid. The presence of water vapor was found to affect the accessibility of the double bond for reaction as well as the reaction probability (*γ* = 6 × 10^–7^). Several studies by Finlayson-Pitts and co-workers have shown that unsaturated phospholipids are readily oxidized by O_3_ from the “top down” to generate secondary ozonides and a number of products.^122–124^ The group of Ye^125,126^ probed the structure and stability of phospholipids in low ozone concentration by a combination of π–A isotherms, vibrational spectroscopy, and force microscopy. Their results demonstrate that the C=C moieties in the unsaturated lipids are selectively oxidized by a trace amount of ozone in the ambient environment. The oxidation process, however, is partially inhibited by mixing with the saturated lipids in the monolayers. This information is useful to understand the stability and functionality of cell membranes under similar conditions.

**Fig. 5:**
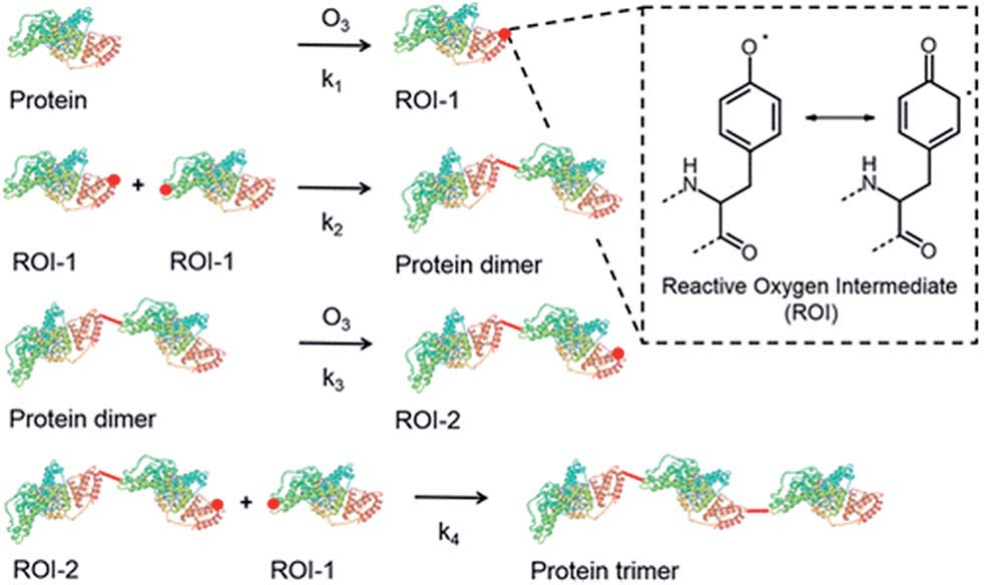
Schematic overview of the most relevant reactions and intermediates for protein oligomerization upon exposure of protein to environmentally relevant O_3_ concentrations. Reprinted from Kampf *et al.*, *Environ. Sci. Technol.*, 2015, **49**, 10859–10886.

Ozone is also known to be a notorious air pollutant. The US Environmental Protection Agency (EPA) concluded that ozone pollution poses serious health threats ranging from productive and development harm to early death.^127,128^ Chemical interactions of ozone with protein are shown to induce post-translational modifications, similar to nitration, rendering the altered protein more allergenic than the original.^99^ A number of studies investigated the general mechanisms and kinetics of protein exposure to O_3_ as well as protein nitration.^104,105,129^ Protein exposure to ozone leads to formation of dimers^105^ and oligomers^130^ which may also proceed through the tyrosyl radicals as shown in Fig. 5. The chemical mechanism for the dimerization of protein such as bovine serum albumin (BSA), a globular protein with molecular mass of 67 kDa and 21 tyrosine residues per molecule, by O_3_ proceeds through a two-step mechanism, as suggested in previous studies.^105^ The first step involves the formation of long-lived reactive oxygen intermediates (ROI-1) from the reaction of a Tyr residue of the protein backbone with O_3_. In the second step, the ROI-1 reacts with each other to form protein dimers. A dimer itself can react further with O_3_ forming tyrosyl radicals, yielding a second type of reactive oxygen intermediate (ROI-2), which may react with ROI-1 to form a trimer (R5)–(R11).^130^

R5 O_3_ + BSA → *c*_1_ROI-1 + (1 – *c*_1_) oxidized monomer

R6 ROI-1 + ROI-1 → dimer

R7 Dimer + O_3_ → *c*_2_ROI-2 + (1 – *c*_2_) oxidized dimer

R8 ROI-2 + ROI-1 → trimer

R9 Trimer + O_3_ + BSA → *c*_3_ROI-3 + (1 – *c*_3_) oxidized trimer

R10 ROI-2 + ROI-2 → tetramer

R11 ROI-1 + ROI-3 → tetramer

where *c*_1_, *c*_2_, and *c*_3_ are stoichiometric coefficients for (R5), (R7), and (R9) respectively. The reaction rate of ozone uptake of BSA showed an upward trend with increasing relative humidity, which can be ascribed to an increase of bulk diffusivity of O_3_ due to the transformation of the glass-like, amorphous protein, in which diffusion is slow, to more liquid-like substrates.^129,131–133^

Geddes and coworkers studied the transformation of dipeptides in an unsaturated organic matrix (*i.e.*, oleic acid) upon exposure to ozone. Importantly, their results showed that peptide groups that do not feature side groups susceptible to direct ozonolysis may still undergo chemical transformations within the particle as a result of reactions with the ozonolysis products of the unsaturated components. Using photoelectron resonance capture ionization aerosol mass spectrometry (PERCI-AMS), they found that upon exposure to ozone, mixed particles containing a dipeptide and oleic acid resulted in the loss of peptide ions and carrier matrix and the appearance of ion signals corresponding to high molecular weight imides and amides. This type of reactivity is hypothesized to be of importance for aerosols containing cell derived materials including proteinaceous matter and unsaturated components such as fatty acids, phospholipids, and other unsaturated lipids.^134^

It is known that the viability of bacteria on surfaces is directly affected upon exposure to ozone.^135^ Furthermore, it was found that the absorption and fluorescence of aromatic amino acids in solution^136,137^ and in animal tissues^138^ changed upon ozone exposure. However, the effects of ozone on the fluorescence properties of the bacterial component of bioaerosols have not been extensively studied. Studies on the fluorescence spectra of un-aerosolized pollens treated with ozone were conducted by Roshchina.^139,140^ Effects of ozone and relative humidity on fluorescence spectra of bioaerosol particles have been investigated in a number of studies to simulate atmospheric processing. Santarpia *et al.* ^141^ exposed samples of aerosolized *Yersinia rohdei* and MS2 bacteriophage-in-*Escherichia coli* lysate to atmospheric concentrations of ozone and found that the fluorescence peak at 330 nm decreased in intensity and became slightly blue shifted. Using a rotating drum chamber, Ratnesar-Shumate and coworkers^142^ conducted aging experiments by subjecting bioaerosols, *Bacillus thuringiensis* Al Hakam (BtAH) spores and MS2 bacteriophages to O_3_ and varying RH. The experiments yielded a significant decrease in the fluorescence for both BtAH and MS2 at high ozone concentration and high RH when excited at 263 nm excitation. The decreases in 263 nm excited fluorescence are indicative of hydrolysis and oxidation of tryptophan in the aerosols. Similarly, Pan *et al.* studied the properties of single octapeptide bioaerosol particles when exposed to O_3_ and RH.^143^ The results of these studies indicate that the physical and biological properties of bioaerosols change significantly after exposure to ozone and water vapor. The exact mechanisms for the observed changes were not reported and require future study.

Finally, ozone can dramatically change the structure and functionality of many biomolecules.^144,145^ Studying the effect of ozone exposure at the air–water interface, Hemming and coworkers^146^ found structural damage to the lung surfactant protein B. In addition, they found out that the presence of phospholipid, DPPC does not prevent oxidation of the peptide at the conditions in which the study was conducted. Similarly, ozone exposure caused degradation and rearrangement of lung surfactant phospholipid, POPC.^147^ Novel mass spectrometric approaches have enabled researchers to enquire the interaction of O_3_ with biomolecules at an air–liquid interface. Under conditions relevant to the interaction of ambient O_3_ with lung surfactant, these phase-boundary experiments confirm that cysteine (Cys) residues are the preferential sites of oxidation in peptides and proteins.^148–151^ Cys often serves as the redox active site in peptides and proteins. Recently, Su *et al.* ^152^ reported the valence electronic properties and pH-dependent ionization energies of Cys aqueous bioaerosols using the aerosol VUV photoelectron spectroscopy. Ionization energy determines how readily the electron can be taken away from the donor. Their results show altered molecular orbital character of Cys when its form changes with pH.^152^ Deoxyribonucleic acid (DNA) and ribonucleic acid (RNA) are also prone to the oxidizing potential of ozone.^145,153,154^

### D. Other trace gases, new reaction pathways and photochemistry

Another important trace gas in the atmosphere is sulfur dioxide (SO_2_). Although SO_2_ is known to react with surfaces in mineral dust,^155–157^ its reactive collisions with biological and organic aerosol have not been well explored. Shang *et al.* ^158^ investigated the uptake of SO_2_ on oleic acid (OA), which is a monounsaturated long fatty-acid found in plant membranes that has been detected in atmospheric aerosols.^159^ Their experiments on the heterogeneous uptake of SO_2_ on OA showed a decreasing uptake coefficient with increasing SO_2_ concentration, suggesting a Langmuir–Hinshelwood type mechanism characterized by the adsorption of the oxidant followed by a chemical reaction on the surface or within the bulk. In the presence of water vapor, the uptake coefficient of the reaction revealed an upward trend with increasing RH. Monitoring the products of the heterogeneous reaction of OA with SO_2_ using high resolution mass spectrometry (HRMS) revealed that, devoid of any other oxidants, sulphur-containing compounds were produced, leading the authors to conclude the formation of organosulfates.^158^ The direct SO_2_ addition may be relevant in highly populated megacities such as those in China where sulphate levels are high.

**Fig. 6:**
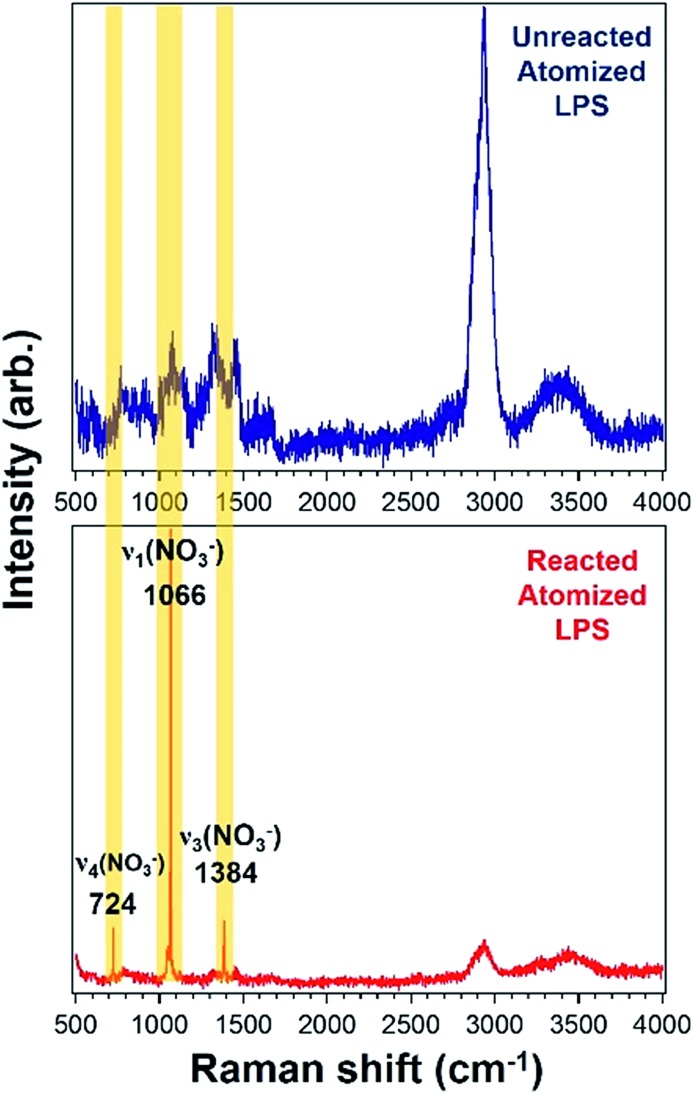
Raman spectra showing reactivity of LPS before (blue) and after (red) exposure to HNO_3_ acid. The yellow highlighted peaks represent the nitrate vibrational modes. Adapted with permission from Trueblood *et al.*, *J. Phys. Chem. A*, 2016, DOI: 10.1021/acs.jpca.6b07023.

**Fig. 7:**
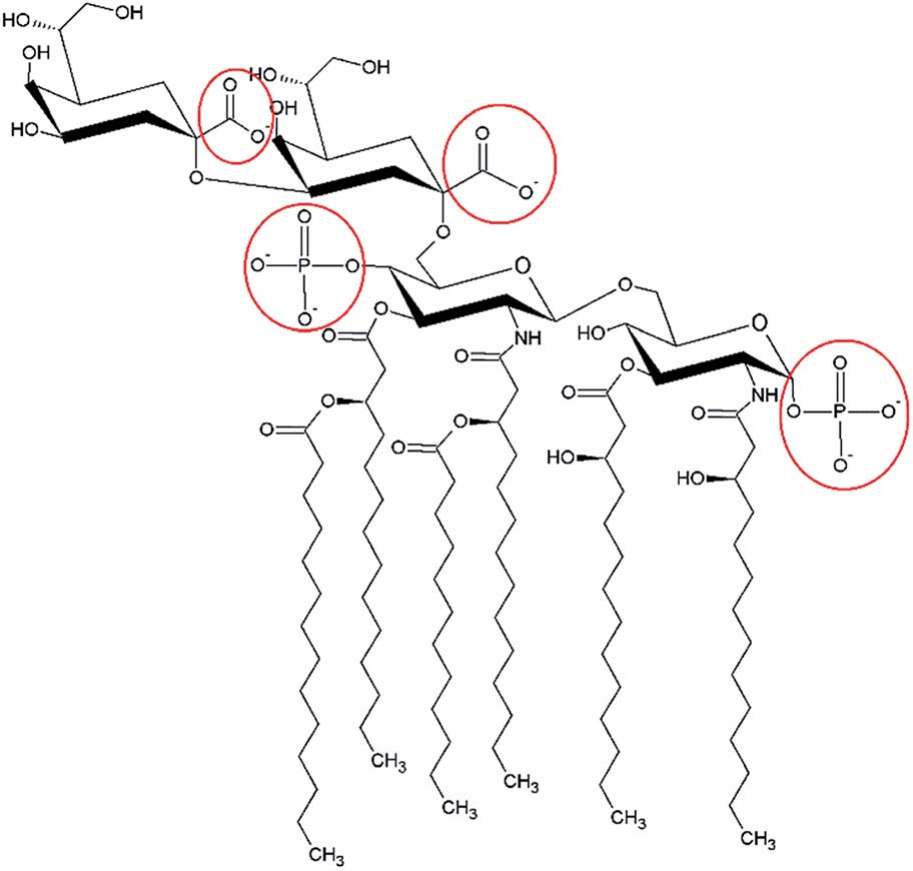
Basic components of lipid A, one of the main components of LPS that could react with gas phase nitric acid. The phosphate and carboxylate sites are highlighted and are found in the lipid A and inner core oligosaccharide regions of LPS.

Another type of reaction in the atmosphere is the acid–base reaction. It has been very well documented in aerosol studies, in both atmospheric and laboratory settings, that atmospheric nitric acid (HNO_3_) can displace chloride in the SSA particles rendering the aerosol deficient in chloride ion content.^51,58^ Single particle analysis on substrate deposited particles collected from a unique ocean-atmosphere facility^160^ show that biologicals exhibit heterogeneous reactivity towards HNO_3_.^161^ Using Raman microspectroscopy, Trueblood *et al.* proceed to show that lipopolysaccharide (LPS), a biomolecule found in the outer membrane of Gram-negative bacteria that has been detected in SSA, does react with HNO_3_ as shown in Fig. 6.^162^ This is manifested by the growth of nitrate peaks (*ν*_4_ 724 cm^–1^, *ν*_1_ 1066 cm^–1^, and *ν*_4_ 1384 cm^–1^) following exposure of atomized LPS to HNO_3_ acid vapor. Highlighted in red, Fig. 7 shows the basic components of the complex structure of lipid A, one of the main components of LPS. The carboxylate and phosphate groups can effectively react with HNO_3_.

Hydrogen peroxide (H_2_O_2_) is a powerful oxidant in anoxic environments. Stored in ice and glacier, it is released directly into the ocean and the atmosphere upon melting and could mediate global oxidation events.^163^ In addition, H_2_O_2_ and organic peroxides play important roles in the cycle of oxidants and the formation of SOA in the atmosphere.^164^ H_2_O_2_ in the presence of ferrous ion (Fe^2+^) produces the OH radical and ferryl iron (Fe^3+^), which is lethal to the cell and other bioaerosols.^165^ Notably, the H_2_O_2_ vapor finds its use in the decontamination of areas prone to accumulate biological pollutants. For instance, the indoor environment can be contaminated with bioaerosol such as viruses, bacteria, fungi, and their spores, either from natural occurring or outdoor environmental origins.^166^ It is also important to note that other oxidants exist in the atmosphere that can induce chemical aging of bioaerosols. Halogen atoms (*i.e.*, Cl, Br, I) formed from marine environments^167^ and less reactive radicals such as HO_2_, ClO, and BrO can drive oxidation of bioaerosols.

Besides the presence of oxidants, there are other stimuli that can perturb the equilibrium chemical and physical state of bioaerosols. Sunlight promotes chemical processes in atmospheric molecular systems substantiated by the presence of photons of the correct wavelengths.^168^ Solar illumination can initiate photochemistry to specific radiation-absorbing chromophores present in the atmosphere^169^ and surface ocean.^170^ Subsequently, this reaction release different classes of organic compounds to the atmosphere. Studies done by Ciuraru *et al.* have demonstrated the production of gas phase functionalized and unsaturated organic compounds resulting from the degradation of surfactants by the photosensitized reactions involving dissolved organic matter.^171–173^ Irradiation with UV light can directly generate a series of biochemical events inside airborne cells like, for example, photo-oxidation of proteins that cause a complex cellular response involving DNA damage, cellular transformation, and eventually cell death.^174^ Indirectly, protein oxidation by irradiation is caused *via* the formation and subsequent reactions of singlet oxygen (^1^O_2_) generated by the transfer of energy to ground triplet state molecular oxygen by protein-bound chromophores.^175^ Sudden shifts in temperature and changes in pH can disrupt metabolic activity of bacteria killing some species and forcing others to adopt several survival mechanisms and adapt to a stressful environment.^19^

## Summary and future outlook

Once airborne, aerosol particles transform rapidly from their freshly emitted states as they are exposed to atmospheric oxidant gases such as OH, NO_3_, and O_3_. Through heterogeneous and multiphase chemistry, these oxidants can significantly modify bioaerosol composition and properties. OH-initiated oxidation of bioaerosols leads to H-atom abstraction, carbon–carbon bond breakage and subsequent formation of smaller molecular species. The ozonolysis reaction proceeds through fragmentation reaction to form several products including gas phase precursors for SOA formation. Nitration of biomolecules scales the protein allergenic potential upwards. The presence of other trace gases can be a path to forming new atmospherically important species like organosulfates. Thus, there are large consequences of heterogeneous and multiphase chemistry of these biologically-derived aerosol particles. Heterogeneous and multiphase oxidation can result in a loss of biomarkers needed to track the formation and chemical transport like, for example levoglucosan and abietic acid which are both important molecular markers of biomass burning aerosol.^176–178^ Besides these chemical transformations, there are changes to the hygroscopicity, which can potentially affect the ability of bioaerosols to be effective cloud and ice nuclei and can thus alter cloud droplet number and size and modify radiative forcing. This can impact the lifetime of a particle in the atmosphere. A summary of select studies on the reaction of atmospheric oxidants with bioaerosols is summarized in Table 1.

**Table 1:** Summary of studies on the reaction of select atmospheric oxidants with bioaerosols

Reaction	Reaction products and rate constant (*k*)	Ref.
OH + DPPC	(Top down) aldehydes, ketones, organic nitrate and nitrite ions; *k*_OH+DPPC_ = (7 ± 1) × 10^–14^ cm^3^ per molecule per s	Dilbeck and Finlayson-Pitts (2013)^83^
OH + POPC	(Top-down) aldehydes, ketones, organic nitrate and nitrite ions; *k*_OH+POPC_ = (3 ± 1) × 10^–13^ cm^3^ per molecule per s	Dilbeck and Finlayson-Pitts (2013)^83^
OH + OPPC	(Bottom-up) phospholipid aldehyde, organic nitrates and carbonyl compounds	Karagulian *et al.* (2008, 2009)^84,86^
OH + GSH	Sulfenic acid (GSOH^–^), sulfinic acid (GSO_2_H^–^), sulfonic acid (GSO_3_H^–^)	Enami *et al.* (2015)^78^
NO_3_ + aromatic dipeptides	Nitroaromatic compounds	Gamon *et al.* (2014)^110^
NO_3_ + aromatic amino acids	Nitroaromatic compounds	Goeschen *et al.* (2011)^109^
O_3_/NO_2_ + BSA	Nitrated tyrosine, protein dimers	Shiraiwa *et al.* (2012),^105^ Franze *et al.* (2005)^89^
O_3_ + OPPC	Secondary ozonide then further decomposed to aldehydes, carboxylic acids and anhydrides; *k*_O_3_+OPPC_ = (4.5 ± 0.6) × 10^–16^ cm^3^ per molecule per s	Karagulian *et al.* (2008)^118^
O_3_ + POPC	Secondary ozonide, phospholipid aldehyde, carboxylic acid	Dilbeck and Finlayson-Pitts (2013)^121^
O_3_ + BSA	Protein dimers, trimers, and higher oligomers	Kampf *et al.* (2015)^130^
O_3_ + dipeptide (dileucine)	High molecular weight imides and amides	Geddes *et al.* (2009)^134^
O_3_ + POPG	Hydroxyhydroperoxide and the secondary ozonide	Kim *et al.* (2010)^148^
O_3_ + cysteine	Cysteine sulfenate (CySO^–^), cysteine sulfinate (CySO_2_^–^), and cysteine sulfonate (CySO_3_^–^)	Enami *et al.* (2009)^151^
SO_2_ + oleic acid	Organosulfates	Shang *et al.* (2016)^158^

The field is complex and truly requires expertise and practitioners from a variety of fields including biology/microbiology, biochemistry/chemistry, atmospheric science, physics, proteomics and genomics. Furthermore, the strength of our current understanding of atmospheric aerosols builds on the collaborative efforts of atmospheric observations, laboratory investigations, and computer modeling. In particular, the increase in the number of observations in recent times in parallel with careful experiments in the laboratory shows a massive acceleration in the interest of probing the complex nature of primary bioaerosols and biologically derived particulate matter, so much so that the perspective given in this article only represents a select summary. Our understanding is far from complete and a significant effort is needed to guide this science from:

(i) A greater understanding of the production mechanisms of bioaerosols,

(ii) Understanding the composition, structure and reactive sites in bioaerosols,

(iii) Identifying reaction pathways involving trace gases with bioaerosols with a focus on oxidant chemistry, acid–base chemistry and,

(iv) Integrating this information and data to improve our quantitative understanding at the process level of how bioaerosols interact with trace gases and how this alters their impacts on health and climate.

As researchers proceed to define new reaction pathways for bioaerosols, it is equally important to investigate how solar radiation affects the chemistry of ocean surface water that drives the formation and release of new compounds to the atmosphere and to understand how the interaction with water vapor changes the size, chemical composition and water uptake of these compounds.

Looking ahead, the trajectory of bioaerosol studies in particular will continue to progress in ways we can only anticipate and will constantly pique the inquisitive minds of scientists because “particulate matter matters”.^179^ Atmospheric chemistry a well-established field of study now gives rise to atmospheric biochemistry which is in its infancy poised to open up a new set of scientific opportunities and challenges.
